# Unhealthy Alcohol Use in People Living with HIV: A Narrative Review

**DOI:** 10.1007/s11904-026-00799-8

**Published:** 2026-10-29

**Authors:** Samuel Kizito, Fred M. Ssewamala, Shaddy K. Saba, Proscovia Nabunya, Nhial Tutlam, Massy Mutumba

**Affiliations:** 1 Silver School of Social Work, New York University, New York, NY 10003 USA; 2 School of Public Health, Washington University in St. Louis, St. Louis, MO 63130 USA; 3 Silver School of Social Work, McSilver Institute for Poverty Policy and Research, New York University, New York, NY 10003 USA

**Keywords:** Alcohol use disorder, Unhealthy alcohol use, HIV/AIDS, Antiretroviral therapy, Integrated interventions

## Abstract

This narrative Review provides a synthesis of the current evidence on the epidemiology, mechanisms, and interventions for unhealthy alcohol use (UAU) in people living with HIV (PLWH). UAU and HIV form a syndemic relationship that negatively impacts HIV treatment outcomes, including reduced antiretroviral therapy (ART) adherence and viral suppression, and increased mortality. Despite the high prevalence rates of UAU among PLWH, screening and treatment gaps persist in this population. Behavioral factors—such as intentional ART skipping and condomless sex—and structural barriers, including poverty and alcohol-related stigma, further worsen HIV outcomes. Moreover, underutilized screening tools and provider hesitancy continue to hinder alcohol treatment for PLWH. Consequently, UAU remains underdiagnosed in PLWH, while those diagnosed are less likely to receive treatment. Promising interventions such as pharmacotherapy (e.g., naltrexone), cognitive behavioral interventions (e.g., motivational interviewing and contingency management), and combination economic empowerment programs (e.g. conditional and non-conditional cash transfers) are currently inconsistently implemented. Action is urgently needed to implement and standardize UAU screening and management in PLWH. Addressing this syndemic phenomenon demands a holistic approach combining medical/clinical, behavioral, and structural solutions to improve survival and quality of life for PLWH.

## Introduction

 HIV and Unhealthy Alcohol Use (UAU) rank among the top pressing public health challenges worldwide [1, 2]. UAU, defined as alcohol consumption that increases risk of health consequences [3], encompasses a spectrum that includes, on one end, less severe consumption patterns—such as drinking greater than weekly or daily recommended limits (i.e., heavy drinking or binge drinking)—which can also have health consequncesconsequences and progress to AUD; and on the other end, clinically severe alcohol use disorder (AUD), defined as a problematic pattern of alcohol use that results in the inability to control or stop alcohol use, leading to significant health and psychosocial consequences [4–6]. For people living with HIV (PLWH), UAU results in poorer HIV treatment outcomes, including reduced antiretroviral therapy (ART) adherence, accelerated HIV progression, increased HIV transmission risk, and mortality [7, 8]. Despite the substantial research linking UAU to worse outcomes in PLWH, as was documented in a scoping review by Hahn and Samet [8], unhealthy use continues to undermine the HIV care continuum. The consistently poor outcomes in this population indicate persistent knowledge and practice gaps, warranting special attention to UAU in the context of HIV. This review article synthesizes the current evidence on the epidemiology, mechanisms, and interventions for UAU in PLWH. The review also highlights key knowledge gaps and care deficiencies and discusses strategies to address the identifiable gaps.

An estimated 39 million people are living with HIV globally, with sub-Saharan Africa (SSA) bearing the highest burden, accounting for 67% of all HIV cases [1]. Annually, there are more than 1·3 million new cases of HIV, with 630,000 HIV-related mortalities [1]. Concurrently, over 280 million individuals meet criteria for AUD diagnosis—the severe form of UAU—worldwide, and alcohol is responsible for 3·3% of all HIV-related deaths annually [2]. Among PLWH, the AUD prevalence is 2–4 times higher than in the general population, estimated at 20% − 50% [9].

Geographical variations exist in the prevalence of alcohol consumption and UAU among PLWH, with developed countries reporting higher levels than low- and middle-income countries (LMICs). Duko and colleagues conducted a meta-analysis of 25 studies comprising 25,000 PLWH to examine AUD prevalence and reported 42·1% prevalence in developed countries compared to 24·5% in LMICs [9]. In SSA specifically, lower but varying AUD prevalences have been reported. Necho and colleagues summarized the AUD prevalence in PLWH from SSA in a recent meta-analysis of 22 studies, reporting a pooled AUD prevalence of 22·0%. Higher prevalences were reported in South Africa (28·8%) and Nigeria (22·8%), while other countries, such as Uganda, reported slightly lower prevalences (16·6%) [10]. Notably, both alcohol consumption generally and UAU specifically have been increasing in LMIC in the past several decades and are projected to continue increasing through 2030 [11].

Among PLWH in the United States, a 2016 systematic review of 113 studies reported a prevalence of UAU of 24% [12], while a more recent study (2021) reported a 32% prevalence of binge drinking with some regional variation [13]. In Eastern Europe, the numbers for UAU among PLWH are equally high. For example, a recent community-based study in Russia reported a 30% rate of binge drinking [14], and another study reported a 60% rate of unhelahty alcohol use in the past 30-days prior to the study [15]. The high prevalence of AUD in Eastern Europe is concerning, since the region accommodates over 1.4 million PLWH and is facing the fastest increase in the HIV epidemic in the world, with over 48% increase in HIV incidence in the last decade [16, 17]. As only a handful of studies explore behavioral or biomedical interventions for UAU among PLWH in Eastern Europe—primarily in Russia [18, 19]—this is a critical area of additional research.

In Asia, available studies report substantial but heterogeneous burdens of UAU among PLWH, although estimates are difficult to compare directly because the studies rely on different definitions and measurement of alcohol use. For example, one study from India reported that 31% of men LWH met criteria for AUD, whereas a study from Thailand found a 14% prevalence of UAU, and 7.9% prevalence of AUD [20, 21]. In South America, Brazil has reported high levels of UAU among PLWH, with one study among PLWH at a referral hospital documenting that 28.6% screened positive for UAU and 5% met the criteria for AUD [22]. These figures should be interpreted cautiously, as most studies draw from clinic-based samples and use varying thresholds and instruments, likely underestimating the true population burden and limiting direct regional comparisons.

Certain subpopulations of PLWH are known to be particularly vulnerable to UAU, with female sex workers, men who have sex with men (MSM), people who inject drugs (PWID), and youths living with HIV being among the groups who are especially at risk [23, 24]. Not surprisingly, these vulnerable HIV populations frequently encounter barriers to care due to factors such as discrimination and criminalization [25]. It is critical to develop and study interventions designed to reduce UAU and support safer drinking patters in these vulnerable populations, given the evidence associating UAU with poorer adherence to ART [26] and other HIV treatment outcomes.

Another challenge to addressing UAU in PLWH is the underutilization of the available effective strategies in routine clinical practice for this population [27]. There is evidence PLWH are less likely to be screened for UAU, and those with identified problems are less likely to receive treatment [27, 28]. For example, in a cohort study of over 800,000 outpatients who screened positive for UAU across 1039 Veterans Health Administration clinics in the United States, PLWH were 20% less likely than HIV-negative patients to receive brief intervention or any alcohol-related care [27]. Without additional efforts, UAU will continue to undermine HIV outcomes, including desired prevention and treatment outcomes.

## Unhealthy Alcohol use Mechanisms in HIV

### Immune System

**Fig. 1:**
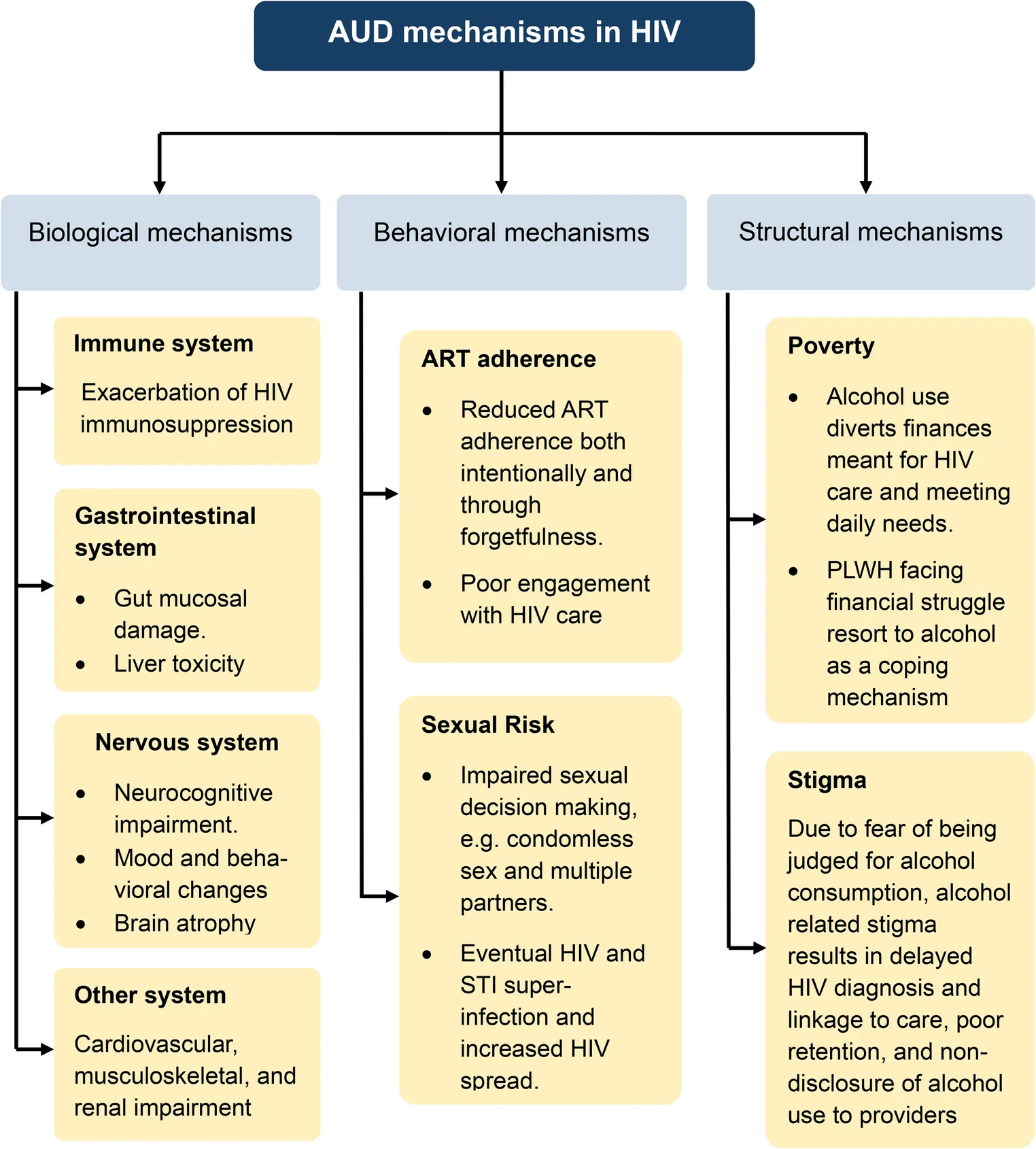
Mechanisms underlying the influence of alcohol-use disorder on HIV

Alcohol consumption worsens HIV prognosis in various ways, one of which involves weakening the immune system (Fig. 1). Chronic alcohol use disrupts both innate and adaptive immunity, exacerbating HIV-associated immunosuppression, increasing susceptibility to opportunistic infections (e.g., tuberculosis and pneumonia), and increasing HIV replication [29]. A detailed account of the effect of alcohol on the immune system was published in a special issue (2015) of *Alcohol Research: Current Reviews* dedicated to alcohol and immune function [30]. Alcohol disrupts the immune system in various ways. At the cellular level, alcohol can impair the function of B and T lymphocytes, natural killer (NK) cells, and macrophages—leading to depressed CD4 + T-cell counts, cytokine imbalance, and impaired T-lymphocyte activation [31]. A more recent meta-analysis (2021) of 23 studies on inflammatory cytokines and AUD found that interleukin 6 (IL-6) was consistently higher among patients with AUD than among healthy controls [32], indicating its key role in immune modulation in this group. Additionally, alcohol use is associated with micronutrient deficiency, which further impairs the immune system [30].

### Gastrointestinal (GI) System

Alcohol can alter the GI microbial homeostasis and impairs the GI mucosa. A 2025 systematic review of 91 articles reported that alcohol and its metabolites damage the tight junctions between gut mucosal cells, resulting in mucosal barrier dysfunction [33]. This damage to the GI mucosa facilitates microbial translocation, inducing systemic inflammation, mediated through T cell activation and production of interferon-gamma (IFNG) and tumor necrosis factor (TNF). The systemic inflammation resulting from the alcohol induced GI mucosal disruption contributes to alcohol-induced liver disease (ALD) [33]. Hence, severe or long-term AUD results in fatty liver and cirrhosis. The impaired liver function results in a harmful accumulation of HIV drugs that can further damage the liver [34].

Alcohol consumption also interferes with ART metabolism in the liver. Through induction of cytochrome P450 enzymes—particularly CYP3A4—chronic alcohol use alters the metabolism of several antiretroviral agents, including certain protease inhibitors and non-nucleoside reverse transcriptase inhibitors (NNRTIs), potentially leading to sub-therapeutic or highly variable drug levels [34]. In addition, alcohol-related liver injury may compound the hepatotoxicity associated with some antiretroviral medications, such as nevirapine [35]. Although older nucleoside analogues such as Didanosine and Stavudine are no longer used in contemporary HIV treatment, their historical toxicity emphasizes the vulnerability of the liver in PLWH with UAU [36]. Although current first-line regimens are substantially safer, individuals with advanced liver disease—particularly those with alcohol-associated liver injury—still require careful ART regimen selection and close monitoring. A small number of studies have examined ART management strategies in PLWH with co-occurring UAU and liver disease, including observational work on integrase inhibitor–based regimens and limited trials evaluating hepatology-HIV co-management models [37, 38]. However, rigorous intervention studies tailored specifically to this population remain scarce, representing an important gap for future research.

### Nervous System

The synergistic neurotoxicity of alcohol and HIV precipitates more severe and rapid cognitive impairment than either condition alone [39]. Within the central nervous system (CNS), chronic alcohol exposure induces neurocognitive damage via dysregulation of glutamatergic and dopaminergic signaling in the cortico-striatal-limbic system, a pathway implicated in both addiction and HIV-associated neurocognitive disorders [40]. Even at low blood levels (< 0·7%), alcohol directly impairs cognitive functioning through memory deficits and executive dysfunction, which can disrupt medication adherence [41]. While some studies report domain-specific alcohol- and HIV-related CNS deficits such as verbal IQ and semantic memory, others demonstrate global cognitive deterioration [42, 43]. Proposed mechanisms include chronic neuroinflammation, microglial activation, and cumulative ART neurotoxicity [39].

### Unhealthy Alcohol Use and Older Age

Although the limited available literature shows a gradual reduction in unhealthy alcohol consumption with increasing age, older PLWH have been shown to have higher rates of UAU than those without HIV of comparable demographics [44, 45]. Understanding UAU and its effects in older PLWH is particularly important as HIV is increasingly considered a chronic condition as more PLWH survive to old age. For instance, 43% of PLWH in the US are above 54 years.

Older PLWH exhibit markedly worse alcohol-related sequelae compared to their young counterparts [39]. The exaggerated effects of alcohol among this age group can be explained by various mechanisms, such as the slower metabolism, comorbidities, and polypharmacy, some of which may interact with alcohol or ART to exacerbate the poor treatment outcomes [46]. Satre and colleagues (2025) conducted a systematic review of 14 studies in the United States and found that among older PLWH, those with greater alcohol consumption are more vulnerable to frailty [44].

However, there is scant large-scale epidemiological data on the burden of UAU, specifically among elderly PLWH, with the exception of isolated Veteran Affair (VA) cohort studies in the United States [47]. Additionally, though some alcohol treatment trials among PLWH have included substantial proportions of older participants [46], we found no trials that were explicitly designed and tailored for older PLWH and UAU. Existing intervention studies either aggregate older PLWH with younger adults or focus on aging with HIV more broadly, with substance use addressed only as one of several comorbidities rather than as a primary treatment target [5]. This lack of age-specific interventions is striking given the growing number of older PLWH globally, and the distinct clinical, psychosocial, and pharmacologic challenges they face in the context of UAU.

### ART Adherence

Alcohol consumption is strongly associated with reduced ART adherence. Our literature search identified several systematic reviews on alcohol use and ART adherence among PLWH [41, 48, 49]. While the definition of alcohol use and ART adherence varied across these studies, AUD consistently predicted poor ART adherence. For instance, Vagenas et al. reviewed 53 studies published between 2010 and 2015 and found that 77% of the studies reported a negative impact of alcohol consumption on the HIV care continuum [25]. More recently, Ge and colleagues reported similar results in a subgroup analysis of 13 studies involving PLWH, drawn from their systematic review of 47 studies [50].

The lower ART adherence in PLWH and co-occurring UAU is driven by both intentional decisions and unintentional factors, e.g., forgetfulness while under the influence of alcohol. Alcohol use can directly disrupt HIV treatment through deliberate ART skipping or discontinuation, often due to toxicity concerns [41]. A landmark study by Kalichman et al. revealed that up to 80% of PLWH who drink alcohol intentionally skipped ART doses to avoid perceived alcohol-ART interactions and toxicities [51]. These findings reflect a prioritization of alcohol use over HIV treatment adherence and call for more efforts to address AUD in PLWH. Importantly, these associations do not imply that PLWH who use or misuse alcohol should be withheld ART. Even in the context of UAU, ART remains highly effective, saves lives, and can achieve high rates of viral suppression when adherence is adequately supported.

### Alcohol Use Motivations, Stress, and Behavioral Health

Alcohol use among PLWH is frequently motivated by coping with HIV-related stress, including stigma and economic hardship [52]. These psychological and structural factors create a cyclical relationship, where alcohol use worsens stress and undermines HIV management, while stress perpetuates hazardous drinking. Therefore, UAU often coexists with other mental health disorders, such as depression, creating additional barriers to engagement with treatment for PLWH [53, 54]. Similarly, UAU often co-occurs other substance use as reported in a recent, large epidemiological study of PLWH, in which over 9% of those with UAU also exhibited unhealthy use of other substances (e.g., cannabis, stimulants, opioids) [55]. This co-occurrence of UAU with other morbidities calls for integrated care models. Indeed, a scoping review of 29 articles found that integrating HIV and mental health services often led to reductions in alcohol use or AUD among PLWH [56].

However, the integration of alcohol use and mental health services within HIV care varies substantially across region and health system. In the United States, models—such as the VA and Ryan White HIV/AIDS Programs—have established integrated behavioral health and HIV care services [57]. In contrast, similar integrated care models remain far less common in LMICs, although a few notable programs exist. For example, pilot programs in Kenya and Uganda have tested the incorporation of brief alcohol interventions, mental health screening, or task-shifted counseling within HIV clinics, demonstrating feasibility but limited scalability [58, 59]. These isolated studies highlight both the potential and the persistent gaps: while integrated care is possible in LMIC settings, widespread implementation and sustained system-level integration remain rare. Addressing these global disparities will require expanded investment, workforce development, and policy commitment to integrated care strategies, particularly in regions with high burdens of HIV and UAU.

### Sexual Risk

Alcohol intoxication impairs sexual decision-making, compromising condom use skills, attitudes, and ability to negotiate safer sex—key factors in HIV transmission [60]. In a systematic review on alcohol use and behaviors that increase HIV spread and STI risk (e.g.,condoless sex) among PLWH, Scott-Sheldon et al. reported that only 67% of PLWH used condoms during their last sexual encounter, and alcohol use was significantly associated with unprotected sex [61]. Beyond increasing horizontal HIV transmission, risky sexual behavior among PLWH raises the risk for STI and HIV superinfection—risking the acquisition of resistant HIV strains and complicating HIV management [62].

### Structural Mechanisms

PLWH, especially in SSA, often face psychosocial challenges, notably poverty and HIV-related stigma, which hinders access and adherence to HIV treatment [63]. This syndemic is exacerbated by UAU, creating additional barriers to HIV care and treatment. Alcohol is associated with household financial instability, perhaps through diverting household financial resources [64]. For PLWH and UAU, alcohol consumption may be prioritized over healthcare expenditures, further hindering access to HIV treatments, especially in resource-constrained settings, where competing survival needs often take precedence over disease management. Moreover, PLWH struggling with poverty and financial instability may resort to alcohol use as a coping mechanism [65].

On the other hand, alcohol-related stigma often leads to shame and fear of judgment within healthcare settings, discouraging individuals from seeking HIV testing, which in turn contributes to delayed HIV diagnosis and postpones timely access to care [66]. Furthermore, alcohol-related stigma has been linked with poorer viral suppression among PLWH [67].

Additionally, the neighborhood alcohol environment, which is often linked to poverty, has been conceptualized to increase the risk of HIV through multiple mechanisms [68]. First, a higher density of alcohol outlets in one’s neighborhood leads to increased alcohol consumption, which correlates with a higher frequency of behaviors such as condomless sex, multiple sexual partners, and drug use. Second, it has been found that alcohol outlets influence social and sexual networks by either expanding or contracting social capital, affecting community interactions and norms [68].

Overall, alcohol use has detrimental effects on the care and overall survival among PLWH. These effects were demonstrated in a simulation cohort study where alcohol reduced life expectancy by more than two years (from 21.7 years to 19.6 years) when alcohol was taken in relatively small amounts and up to six years for those with UAU (from 16.1 years to 9.7 years) [69].

## Screening for Unhealthy Alcohol Use

A clinical diagnosis of AUD is made by a trained clinician using Diagnostic and Statistical Manual (DSM-5) criteria [70]. Screening for possible risk for clinically severe AUD, as well as the broader spectrum of UAU, can be done using one of several screening tools. Screening for UAU is recommended for all PLWH, followed by medical and/or behavioral interventions matched to clinical severity and needs [5, 71]. Several screening tools have been developed for alcohol screening in clinical settings [71, 72]. The Alcohol Use Disorders Identification Test (AUDIT) is a 10-item questionnaire that assesses the frequency and quantity of alcohol consumption, binge drinking, lack of control over use, and negative consequences of use [72]. Although AUDIT is not itself a diagnostic instrument, item content and recommended cut-offs (e.g., scores ≥ 8 for men and ≥ 7 for women) are calibrated to identify patterns of alcohol consumption and related harms that correspond to increased likelihood of meeting DSM-5 criteria for AUD; higher scores reflect greater symptom burden and risk of clinically severe disorder. A shorter version of the AUDIT scale, known as the AUDIT-C, was developed to make alcohol use screening more practical [73, 74]. Specifically, the AUDIT-C uses three questions, including [3] * How often did you have a drink containing alcohol in the past year?* [4] *On days in the past year when you drank alcohol, how many drinks did you typically have?* and [5] *How often did you have 6 or more drinks on one occasion in the past year?* AUDIT-C has demonstrated high sensitivity and practicality for rapid identification of UAU, including among PLWH [73, 74].

Another screening test, the Alcohol, Smoking, and Substance Involvement Screening Test (ASSIST) was developed by the WHO to screen for unhealthy use of a wide range of substances, including alcohol [75]. ASSIST risk categories were derived to reflect probability of meeting DSM-defined substance use disorders: moderate- and high-risk scores for alcohol or other substances signal that a more detailed clinical assessment using DSM-5 criteria is warranted and that brief intervention or specialty treatment may be indicated. The ASSIST screening tool consists of eight structured questions that are used to generate a risk score for each substance, and categorizes individuals into low, moderate, or high risk. Since the tool captures other substances besides alcohol, it is particularly valuable for PLWH, who may have co-occurring polysubstance use, as this impacts ART adherence and treatment outcomes. The ASSIST screening tool is reliable, culturally adaptable, and practical for routine screening [75].

A simpler approach to UAU screening involves asking a single-item screening question (e.g., “How many times in the past year have you had 4 (women) or 5(men) or more drinks in a day?”) [76]. This screening question has been validated in primary care settings and can be asked at the time of collecting other patient information, offering an efficient method for initial risk assessment. In this approach, reporting one or more such heavy-drinking days in the past year (4 or more drinks in a day for women, 5 or more for men) is considered a positive screen for unhealthy alcohol use and should prompt further assessment or brief intervention.

A challenge facing the above screening tests is the reliance on self-report, which is vulnerable to recall and social desirability biases, and may underestimate alcohol consumption [77, 78]. Moreover, it is particularly challenging to accurately assess alcohol use for people who consume non-commercially produced alcohol, such as homebrew, which are common in many LMICs. Compared to commercially produced alcohol, homebrew has an unknown but often higher alcohol content, and the users are less likely to disclose its consumption to the healthcare providers [79]. A Kenyan case study reported that 36% of all adults in Kenya consume homebrewed alcohol, which is less costly and more readily available [80]. Furthermore, the accurate quantification of the amount of alcohol consumed is further complicated by the use of non-standardized containers and communal drinking practices, such as drinking from communal pots, which are common in many low-income settings. These challenges call for more reliable means to assess alcohol consumption.

Biomarkers, such as Urine ethyl glucuronide (EtG) and phosphatidylethanol (PEth), have been identified to potentially assist in measuring alcohol use; however, they are rarely used in routine clinical care due to the associated shortcomings. Urine EtG is a relatively affordable biomarker that can adequately detect alcohol consumption for the past day, making it a potential candidate for very recent alcohol drinking screening [81]. However, EtG testing is limited by its inability to detect longer-term or chronic alcohol use, susceptibility to false positives from incidental alcohol exposure (e.g., hand sanitizers, mouthwash), and uncertainty about the standard cutoffs for different populations [82]. Therefore, when used, EtG results should be interpreted with caution and ideally combined with self-report or other biomarkers for better accuracy [82]. Blood PEth levels have been shown to correlate with quantity of alcohol use over a two week period, however, there has been high variability in its performance and in identifying an accurate cut-score for unhealthy use across studies [82]. PEth use is also limited by its relatively high cost [83], the need for specialized laboratory assays, and long turnaround time [84]. Streamlining PEth processing or developing rapid alternatives remains a priority to improve its practical utility for screening and monitoring alcohol use among PLWH. Besides EtG and PEth, additional alcohol-related biomarkers have been evaluated, though most have demonstrated limited sensitivity, short detection windows, or inconsistent performance across populations. A recent scoping review identified ten serum biomarkers under investigation for detecting alcohol consumption, but none have shown sufficient reliability for routine clinical use [85]. The biomarkers differ significantly in their detection window and are susceptible to confounder, limiting their specificity for alcohol. For instance, Cholesteryl Ester Transfer Protein, can detect alcohol use within 24–48 h of consumption, while Total Serum Sialic Acid (TSA) can indicate alcohol consumption over several weeks. However, like other biomarkers discussed in this narrative review, both biomarkers have limited sensitivity and specificity and are influenced by factors unrelated to alcohol use. Given these constraints, any positive biomarker result should prompt a comprehensive clinical assessment—such as the DSM-5 AUD symptom checklist in a clinical interview—to better characterize the patient’s drinking patterns and determine the presence of AUD. Taken together, the limitations facing biomarkers for detecting UAU highlight the need for continued research to develop more accurate, affordable, and scalable biomarkers, for use in PLWH. As more promising biomarkers emerge, efforts will also be needed to streamline laboratory processes and integrate these biomarkers into routine HIV care.

Despite the availability of the various screening tools, screening for UAU is inconsistently performed for PLWH. Moreover, even when done, screening often does not follow appropriate protocols. For instance, a study by Chander et al. found that only 10% of clinicians screened for alcohol use with a standardized tool [86]. Factors such as low provider knowledge and extra workload have been identified as barriers to UAU screening in HIV care [27]. To better support HIV care integration and facilitate improved clinical decision-making, there is a growing need for improving screening practices in these settings.

Furthermore, even when performed, routine screening may fail to identify PLWH with UAU. One challenge is the lack of consistency measuring alcohol consumption across studies and clinical settings. Some assessments are based on the quantity of alcohol consumed over a specified period [87], whereas others focus on drinking frequency or binge-drinking episodes [83]. These differences complicate the interpretation of screening results, contribute to variability in unhealth alcohol use estimates, and make it difficult to establish standardized thresholds for unhealth alcohol use that can be reliably applied in routine HIV care. Collectively, there is a need for consistency in how UAU is measured, not only to strengthen research comparability but also to ensure that evidence can reliably be translated into clinical practice.

## Interventions for Unhealthy Alcohol Use among PLWH

### General Principles

 For patients found to screen positive for UAU, clinical guidelines recommend that providers advise them to adopt healthier drinking behavior (i.e., moderating their use) and abstain from alcohol if they have clinically severe AUD [74]. Because abrupt alcohol reduction or withdrawal can be life threatening, guidelines also recommend that providers assess the need for medically managed withdrawal, a topic discussed in more detail in the pharmacotherapy Sect [74]. It is also important to recognize that many patients hesitate to change their drinking patterns; doing so can be very difficult even when they are aware that their consumption is harmful, and patients require multiple treatment attempts and can return to use [88]. Effective clinical conversations about UAU—both during initial engagement and throughout ongoing treatment—draw on the principles of motivational interviewing, a person-centered, non-stigmatizing counseling approach in which providers elicit and strengthen the patient’s own motivation for change and collaboratively develop an individualized plan with negotiated goals aimed towards long-term improvement [89]. A harm reduction framework is also beneficial, in which any reduction of alcohol use or related risk behaviors, even in the absence of full abstinence, is recognized as meaningful progress [5].

A full biopsychosocial assessment is necessary to enable providers to offer multi-modal treatment (i.e., behavioral, psychosocial, pharmacological, and other interventions) when indicated [90] and to determine the appropriate level of care. The American Society of Addiction Medicine (ASAM) Criteria provide the most widely used framework for matching patients to a continuum of treatment settings, from outpatient services to medically managed inpatient care, depending on multidimensional assessment of patients’ clinical severity and needs [91]. Depending on assessed need and service availability, treatment may be delivered in specialty substance use treatment settings, integrated care settings (including, settings that treat HIV and UAU concurrently) [38], or otherwise by coordinating care among multi-disciplinary medical and behavioral health providers as available.

### Behavioral and Psychosocial Interventions

Screening, Brief Intervention, and Referral to Treatment (SBIRT) is a public health framework for the early identification and management of UAU. Often drawing from the motivational interviewing principles described above, in SBIRT, validated screening is followed by a brief intervention for those who are deemed at risk, and referral to specialty treatment for those with more severe presentations like AUD. Brief Alcohol Interventions (BAIs) represent the intervention component of SBIRT, and they consist of short, structured counseling sessions and have shown efficacy in reducing UAU in the general population [83]. However, recent reviews indicate that the overall effects of SBIRT and related brief interventions are modest and inconsistent across settings, particularly when delivered as a single brief encounter [83, 92, 93]. Evidence suggests that BAIs are most effective for individuals whose alcohol consumption exceeds recommended limits but does not meet diagnostic criteria for AUD [94]. Their primary aim is early intervention to reduce hazardous alcohol consumption and mitigate binge drinking episodes. BAIs are less efficient for individuals with clinically severe AUD [95]. Among PLWH, findings remain mixed. Edelman and colleagues reviewed recent evidence on the efficacy of brief interventions for PLWH (Table 1). They found that only three trials demonstrated reductions in alcohol use or improvements in viral suppression [5], a pattern consistent with earlier systematic reviews across diverse PLWH populations [83, 102].

**Table 1:** Studies on the AUD interventions for PLWH

Authors (year)	Country or region	Study design	Sample size and Population	Intervention	Outcome	Key findings
Springer et al. (2017) [96]	USA	Double-blind Randomized Controlled Trial	100 incarcerated PLWH with AUD transitioning to community	Pharmacological: Extended-release naltrexone (XR-NTX) vs. placebo	AUDIT, Timeline Follow back (TLFB), heavy drinking days, composite alcohol score	XR-NTX delayed time to first heavy drinking day in younger PLWH (20–29 yrs; 24.1 vs. 9.5 days, *p* < 0.001). No overall group difference in viral suppression.
Edelman et al. (2019) [38]	USA	4-site Double-blind Randomized Controlled Trial (Project DAWN)	51 PLWH with heavy drinking and < 95% ART adherence; mean age ~ 51; majority Black and male	Pharmacological: Extended-release naltrexone (XR-NTX 380 mg IM monthly) vs. placebo + standardized counseling	Heavy drinking days (TLFB), PEth biomarker, ART adherence, HIV viral load, CD4, VACS Index	XR-NTX significantly reduced heavy and any drinking days (*p* = 0.03), but did not improve ART adherence, viral suppression, CD4 count, or VACS Index.
McGinnis et al. (2020) [97]	USA (Veterans Affairs)	Longitudinal cohort analysis	7,830 PLWH initiating behavioral AUD treatment between 2008–2017; majority male; 60% African American; median age 53	Pharmacological and Behavioral: Behavioral AUD treatment (BAUD) of varying intensity (single visit to intensive) and/or Medication for AUD (MAUD, e.g., gabapentin, naltrexone)	AUDIT-C scores (alcohol use severity) pre- and post-treatment, changes in CD4 count, HIV viral load, ARV adherence,	AUDIT-C scores significantly decreased in higher BAUD intensity groups, indicating reduced alcohol use. MAUD was associated with CD4 increase (detectable VL) and adherence improvement. Greater BAUD intensity was associated with improved CD4, VL, and ART adherence—especially among those with detectable VL.
Chi et al. (2022) [95]	USA	Observational cohort (EHR-based)	312,056 adult primary care patients screening positive for unhealthy alcohol use; majority White (62–63%), male (59–63%), mean age ~ 45–46	Behavioral: Brief Alcohol Intervention (BAI) in primary care (based on Motivational Interviewing)	Change in heavy drinking days, drinking days/week, drinks/drinking day, drinks/week at 12-month follow-up	Receiving BAI resulted in small but significant reductions in all drinking outcomes (e.g., −0.26 heavy drinking days/3 months, *p* = 0.005). Effects were stronger for those exceeding only daily limits, younger patients, and those without AUD. Specialty AUD treatment was associated with larger reductions in drinking.
Williams et al. (2017) [27]	USA (Veterans Health Administration)	Observational cohort (EHR-based)	2101 PLWH with positive AUDIT-C scores (≥ 5), receiving care in VA system; majority older men, 49% with AUD diagnosis	Brief alcohol intervention (advice to reduce or abstain), documented in EHR within 14 days of screen	AUDIT-C at follow-up (9–15 months); resolution defined as AUDIT-C < 5 and ≥ 2 point drop	77% received BI, 61% resolved unhealthy alcohol use, but BI was not associated with resolution (aIRR = 0.96; 95% CI: 0.90–1.02). Suggests limited effectiveness in routine care.
Chander et al. (2015)	USA (Baltimore, MD)	Randomized controlled trial	148 HIV-infected women with hazardous drinking; mean age ~ 45; 86% Black	Behavioral: Two-session brief alcohol intervention (BI) adapted for women with HIV	90-day TLFB for drinking frequency, binge drinking, drinks per drinking day; ART adherence, viral load, sexual risk	BI significantly reduced 90-day drinking frequency (OR = 0.42, *p* = 0.005) and unprotected vaginal sex. No differences in drinks per drinking day, heavy/binge drinking days, ART adherence, and viral suppression. BI was effective for reducing frequency but not intensity of alcohol use.
Papas et al. (2011) [98]	Kenya (Eldoret)	Randomized controlled trial (Stage 1)	75 HIV-infected outpatients (hazardous/binge drinkers), ARV-eligible or initiated	Behavioral: 6-session, culturally adapted, gender-stratified group CBT delivered by paraprofessionals	Percentage of drinking days (PDD) and drinks per drinking days (DDD) via TLFB; abstinence at 30, 60, 90 days; alcohol saliva tests; Clinical Institute Withdrawal Assessment for Alcohol (CIWA-Ar) for withdrawal	CBT significantly reduced alcohol use vs. usual care at 30 and 90 days (PDD d = 0.95, *p* = 0.0002 and DDD d = 0.76, *p* = 0.002). Also, 69% in CBT group reported abstinence at 90 days vs. 38% in control.
Edelman et al. (2019) [38]	USA (5 VA HIV clinics)	Multisite randomized controlled trial	128 PLWH with AUD; mostly male (98%) and Black (79%); not engaged in formal alcohol treatment at baseline	Integrated Stepped Alcohol Treatment (ISAT): Step 1 - Addiction Physician Management (APM); Step 2 - APM + MET; Step 3 - referral to specialty care. Compared to Treatment as Usual (TAU).	Primary: Drinks/week (TLFB, past 30 days at week 24); Secondary: PEth biomarker, % no heavy drinking days, drinks/drinking day, % days abstinent, HIV VL suppression, VACS Index	ISAT increased alcohol med receipt (51% vs. 26%, *p* = 0.004) but no difference in drinks/week at 24 weeks (*p* = 0.11). Improvements in abstinent days, drinking intensity, and viral suppression seen at 52 weeks. Greater ISAT adherence linked to fewer drinks (*p* = 0.017).
Conroy et al. (2024)[99]	Malawi	Pilot Randomized Controlled Trial (2 arms)	78 couples (156 individuals; 96% male index patients) with HIV and heavy alcohol use (AUDIT-C ≥ 4 men/≥3 women)	Economic/Behavioral: 10-session Mlambe (financial literacy + relationship skills + matched savings) vs. enhanced usual care (EUC; brief alcohol counseling)	AUDIT-C, TLFB (drinking days), PEth biomarker, ART adherence (VAS), viral load	Mlambe reduced drinking days (6.8 to 2.1) and AUDIT-C scores (7.5 to 3.1) at 15 months; 100% viral suppression vs. 91.9% in EUC (NS). No between-arm statistical differences in this underpowered pilot.
Chamie et al. (2023) [100]	Uganda	Randomized Controlled Trial (2 × 2 Factorial design)	680 PLWH with latent TB and hazardous alcohol use (AUDIT-C ≥ 4 men/≥3 women + urine EtG+); median age 39, 69% male	Economic: Escalating financial incentives contingent on negative urine ethyl glucuronide (alcohol abstinence) and/or positive urine isoniazid test (adherence)	Non-hazardous alcohol use (AUDIT-C + PEth < 35 ng/mL), > 90% isoniazid adherence (MEMS caps)	Incentives for alcohol abstinence significantly increased the rate of non-hazardous alcohol use vs. control (17.6% vs. 9.9%; aRD = 7.6%, *p* = 0.0025).
Edelman et al. (2022) [101]	USA	Stepped-wedge cluster Randomized controlled trial	3647 PLWH across 4 clinics; 121 with OUD, 126 with AUD, 420 with TUD; mean age 49, 50% Black, 22% Hispanic, 39% female	Implementation Strategy: Multi-component implementation facilitation (academic detailing, learning collaboratives, site-specific data feedback, marketing)	Provision of medication for AUD (from EHR data)	Facilitation significantly increased provision of MAUD (8% to 17%, *p* = 0.009).

aOR – Adjusted Odds Ratio; APM – Addiction Physician Management; ART – Antiretroviral Therapy; AUD – Alcohol Use Disorder; BAI – Brief Alcohol Intervention; CBT – Cognitive Behavioral Therapy; CI – Confidence Interval; EUC – Enhanced Usual Care; FDA – Food and Drug Administration; IDU – Injection Drug Use; ISAT – Integrated Stepped Alcohol Treatment; MAUD – Medication for Alcohol Use Disorder; MET – Motivational Enhancement Therapy; MI – Motivational Interviewing; MSM – Men who have Sex with Men; PEth – Phosphatidyl-ethanol; PLWH – People Living with HIV; RCT – Randomized Controlled Trial; SOC – Standard of Care; SUD – Substance Use Disorder; TAU –Treatment As Usual; TLFB – Timeline Follow-Back; VACS – Veterans Aging Cohort Study; VL – Viral Load; XR-NTX – ExtendedRelease Naltrexone

Notably, BAIs that show benefit often involve multiple sessions rather than a single encounter, suggesting that sustained engagement may be necessary to achieve meaningful behavior change in this population [87]. Overall, the evidence remains inconclusive regarding whether BAIs alone are sufficient to address UAU among PLWH, and the optimal content and structure of sessions for this population is not well established [5]. Even so, BAIs remain attractive in many settings because they are relatively low-cost and can be delivered by trained non-specialist providers through task-shifting, which enhances scalability in low-resourced settings [103, 104]. Their effectiveness, however, largely depends on providers’ willingness and comfort in initiating conversations about UAU, and integrating these discussions into routine HIV care [105].

Cognitive Behavioral Therapy (CBT) offers a more intensive, skills-based approach for helping PLWH reduce UAU and its consequences. CBT focuses on identifying and modifying maladaptive thoughts, emotions, and behaviors that drive alcohol use, while building coping skills and alternative strategies to manage cravings, mood symptoms, and HIV-related stressors [83]. CBT, which targets maladaptive thinking and behavior that can drive alcohol use, has been shown to reduce relapse rates significantly among PLWH [83]. Among PLWH, CBT has been associated with meaningful reductions in drinking; for example, group CBT was found to be effective in reducing alcohol use among PLWH in Kenya, with the intervention reduced the percentage of drinking days from 63% to 25% [98]. CBT elements are often combined with motivational interviewing (MI), a collaborative counseling style that enhances intrinsic motivation and resolves ambivalence about change [89]. MI-informed CBT protocols can be particularly well suited to PLWH, who may experience competing priorities, stigma, and apprehension about stopping alcohol use, by both strengthening motivation and providing concrete tools to support behavior change [5, 83, 102].

Contingency management (CM) represents a distinct behavioral strategy in which patients receive tangible incentives—such as vouchers or small cash transfers—for objective evidence of alcohol abstinence or engagement in alcohol treatment [106]. Recent evidence supports CM interventions for UAU among PLWH. Several studies published in the last five years have found that CM is both feasible and acceptable to patients and staff, especially when incentives are simple, linked to measurable biomarkers of alcohol use, and are co-located with HIV treatment services [38, 107, 108]. A factorial RCT conducted in Uganda found that escalating financial incentives based on recent alcohol abstinence was associated with significantly lower alcohol use among adults with HIV [100]. Patient and staff perspectives suggest that broadening reinforcement targets (e.g., reductions in use or consistent visit attendance) and embedding CM within stepped-care models may further enhance retention and clinical outcomes [108, 109]. Despite these benefits, CM requires dedicated resources and infrastructure for monitoring and incentive delivery, which can limit scalability in many low-resource HIV care settings [106–108]. CM is worth continued examination and development as a promising adjunct to existing treatments for UAU in PLWH. Although CM adds costs related to incentives and monitoring, economic evaluations in high-income substance use treatment settings suggest that voucher- and prize-based CM can be cost-effective, with per-participant incentive budgets typically in the low-hundreds of US dollars over a treatment episode and favorable cost–effectiveness ratios when reductions in substance use are considered; however, these cost structures may still pose substantial implementation challenges in resource-limited HIV care settings [110, 111].

In addition to professionally delivered care, mutual support programs such as Alcoholics Anonymous (AA) and other twelve-step programs are widely used as adjunctive or stand-alone strategies for sustaining recovery, and clinicians sometimes support participation through an approach known as twelve-step facilitation (TSF) [112]. A recent Cochrane review concluded AA/TSF is an evidence-based intervention that is at least as effective as, and in many trials more effective than, other established psychosocial treatments for achieving abstinence, and may also be more cost-effective due to lower health care utilization [112]. However, research on the effectiveness of AA/TSF among PLWH is sparse, and this population has reported barriers to attendance such as having support systems that use substances, homelessness, and Hepatitis C infection [113].

Despite being promising, psychosocial interventions are more effective in addressing UAU when they are delivered in combination with other interventions. For example, a systematic review of 21 studies (*n* = 6,954 participants) found limited efficacy for stand-alone psychological interventions [83]. In contrast, a large-scale study of 7,830 PLWH showed that combined behavior and pharmacotherapy resulted in both improved AUD and HIV outcomes, including ART adherence and viral suppression [97]. A key limitation of psychosocial and behavioral interventions is that the optimal intensity and duration of the interventions remain unclear.

### Pharmacological Interventions

FDA-approved medications—including naltrexone, disulfiram, and acamprosate—have demonstrated efficacy in reducing alcohol consumption [71, 97, 114]. While most studies on naltrexone have been conducted among the general population, a few studies have tested the drug for treating unhealthy alcohol consumption in PLWH. A recent systematic review by Farhadian and colleagues summarized the literature on the efficacy of naltrexone in reducing heavy drinking among PLWH [115] and found the drug to significantly improve both AUD severity and HIV viral suppression but not ART adherence [96]. A major drawback of naltrexone is its interaction with specific ART regimens, such as efavirenz, which can lower the bioavailability and efficacy of both naltrexone and ART. Extended-release naltrexone (XR-NTX) has emerged as a viable alternative when faced with reduced naltrexone bioavailability, and has demonstrated feasibility and effectiveness in reducing heavy alcohol use among PLWH [96]. In addition, XR-NTX was found to significantly improve viral suppression in a study among post-incarceration PLWH in the US [116, 117].

Disulfiram is another drug that is effective in deterring alcohol consumption, however, it carries significant risks for hepatotoxicity [118, 119]. This is important for PLWH as many are at risk or may have pre-existing liver disease—due to HIV, ART, or alcohol use. Although acute liver injury from disulfiram is relatively uncommon, its clinical spectrum ranges from mild elevations in liver enzymes to fulminant hepatitis, and severe cases can be fatal. Disulfiram-induced liver injury is dose-independent and typically represents idiosyncratic hypersensitivity reactions that can occur within weeks of starting the drug [118]. Therefore, the use of disulfiram necessitates rigorous monitoring for hepatotoxicity, which limits its scalability in resource-limited settings.

Acamprosate, a glutamate and GABA modulator, is another drug that the FDA approved to treat UAU in the general population. More recently (2023), another meta-analysis of 118 RCTs involving 20,976 patients with AUD established that acamprosate significantly reduced UAU [120]. However, there is limited evidence on the efficacy, optimal dosing, and safety for treating UAU in PLWH specifically. Moreover, acamprosate is taken up to three times daily, which may complicate regimen adherence for PLWH already managing complex ART schedules [121].

Recent studies have explored the use of non-FDA approved drugs for UAU. However, most of these off-label compounds have also been studied among the general population, with limited evidence for their efficacy in PLWH [122]. A notable non-FDA approved drug that has been studied in treating UAU among PLWH is varenicline, a nicotinic acetylcholine receptor partial agonist originally approved for smoking cessation. An RCT was recently conducted in Russia among 400 PLWH who engaged in risky drinking and daily smoking to compare the efficacy of varenicline, cytisine (another nicotinic partial agonist used for smoking cessation), and standard nicotine replacement in reducing alcohol use and smoking [19]. While the study did not detect differences in the efficacy of varenicline, all three treatments were associated with a rapid reduction in drinking and smoking behaviors. While these results are promising, they also highlight a need for further investigation into the efficacy of varenicline in treating UAU in PLWH. Other agents under investigation for UAU include glucagon-like peptide-1 (GLP-1) receptor agonists such as semaglutide and liraglutide, which were originally developed for type 2 diabetes and obesity and appear to reduce alcohol intake in preclinical and early clinical studies; bupropion, a norepinephrine–dopamine reuptake inhibitor widely used as an antidepressant and smoking-cessation medication; and gabapentin,, an anticonvulsant used off-label to treat alcohol-related withdrawal symptoms, cravings, anxiety, and insomnia [7, 92, 93]. These drugs may be particularly relevant as alternatives for patients with coexisting metabolic or neurological comorbidities, which are common among PLWH.

A general limitation of using pharmacotherapy for UAU is the limited knowledge, confidence, and experience among healthcare providers in prescribing these medications [86]. In one study, Chandler and colleagues found that 77% of the clinicians lacked the confidence in initiating pharmacological treatments for UAU and instead referred patients elsewhere, contributing to delays or gaps in care [86]. This lack of provider preparedness is particularly consequential in HIV care, where competing HIV clinical care demands and limited access to addiction specialists can further impede both HIV and alcohol treatment. Addressing this treatment gap will require targeted provider education and developing clear and simplified evidence-based alcohol treatment prescribing protocols for frontline HIV clinicians.

### Withdrawal Management

In addition to medications that target AUD directly, clinicians should assess whether patients are experiencing or at risk for alcohol withdraw syndrome (AWS), which typically occurs within 4–12 h after abrupt cessation or marked reduction in heavy or dependent drinking [123]. If present, clinicians should also assess the AWS severity, and initiate treatment. Structured outpatient withdrawal protocols—typically involving symptom-triggered or fixed-dose benzodiazepines, with alternatives such as gabapentin for mild to moderate withdrawal—can be implemented effectively outside of specialty addiction services when clear guidance and monitoring are available [124]. This pharmacotherapy is provided in addition to supportive care, and close follow-up to ensure safety during withdrawal management [125]. Nevertheless, withdrawal management remains under-recognized in HIV care, and the available guidelines have been developed largely for the general population, with minimal research among PLWH. Integrating evidence-based withdrawal management into HIV care may reduce unnecessary referrals, prevent HIV treatment interruptions, and facilitate timely initiation of longer-term treatment for UAU.

### Economic Interventions

Neither pharmacological nor psychological interventions can address the full scale of the HIV and UAU syndemic without addressing broader structural determinants. Most interventions consider UAU at an individual level. However, the drivers and consequences of UAU go beyond to involve the family and the broader society. Recent evidence suggests that poverty and financial stress significantly influence AUD [65, 126]. Therefore, interventions that address poverty are critical in enhancing the effectiveness of traditional behavioral and pharmacological AUD treatments. Conroy and colleagues have tested economic empowerment (EE) interventions for diverse groups of PLWH to address UAU. Their recent study in Malawi (Mlambe) evaluated an integrated economic and relationship-strengthening intervention for couples affected by HIV and UAU [127]. The intervention combined financial literacy training, incentivized matched savings accounts, and relationship skills building to reduce alcohol consumption and improve ART adherence. Results showed a reduction in self-reported drinking days (6·8 to 2·1) and AUDIT-C scores (7·5 to 3·1) over 15 months [99]. Notably, couples also showed improved ART adherence (from 63% to 74%) and viral suppression (100% in the intervention arm vs. 92% in the control arm), although statistical significance was limited by the modest sample size of 78 couples [127]. Another RCT by Brathwaite et al. in Uganda tested the combination EE intervention for young PLWH to address alcohol and substance use [128]. The success of EE interventions is twofold: the immediate financial relief reduces stress-related drinking, while the conditional nature of the transfers reinforces health-seeking behaviors.

This small but growing body of evidence shows that EE may be not an adjunct but a central component of UAU care among PLWH. These findings align with broader research from the Suubi studies conducted in Uganda, highlighting the role of EE in addressing HIV-related risks and improving treatment outcomes [129, 130]. By addressing financial instability and relationship dynamics that contribute to UAU—key drivers of alcohol use in low-resource settings—such interventions may offer a scalable strategy to build upon individual-level strategies. HIV care frameworks should consider integrating EE interventions to achieve more substantial impact.

### Provider-Focused Interventions

Several studies have demonstrated that provider-focused interventions—such as implementation facilitation (ongoing support to help clinics adopt evidence-based care), targeted education, audit and feedback, and clinical decision support—can enhance the provision of evidence-based treatment for UAU within HIV clinics [109, 131]. To illustrate, a large RCT involving 3647 PLWH from 4 HIV clinics in the United States found that implementation facilitation led to a tangible increase in the delivery of medications for AUD over time [101]. Embedding such provider-focused interventions into HIV care systems is crucial for closing the persistent treatment gap and enhancing UAU treatment outcomes.

### Integrated Care Models and Combination Interventions

Despite the beneficial impact of integrated care on HIV and alcohol treatment outcomes, HIV clinics often lack integrated screening and treatment programs for UAU, leading to missed opportunities for early intervention [38]. Integrated care models are critical, given that siloed treatment approaches often fail to engage PLWH with AUD, who face multiple competing priorities and systemic barriers to care. Given the high prevalence of co-occurring mental health disorders and polysubstance use among PLWH with UAU, integrated care models should address these conditions concurrently rather than in isolation [53, 54]. However, current HIV care models frequently under-prioritize alcohol screening, and similarly, addiction treatment programs rarely incorporate HIV-specific considerations. As such, while AUD affects approximately 20–30% of PLWH in many cohorts, fewer than 15% receive appropriate AUD interventions [83]. National policy, particularly in regions with high HIV burdens, should prioritize integrating UAU screening into routine HIV care to ensure that evidence-based interventions no longer remain inaccessible to many PLWH.

## Conclusions and Future Directions

In summary, the available evidence strongly suggests a link between UAU and poor outcomes for PLWH. While progress has been made toward addressing the syndemic, more effort is still required. First, more work is required to integrate treatment for both conditions. However, HIV clinic workflows often fail to address alcohol use systematically. Second, pharmacological interventions, such as naltrexone, show promise but require adaptation for PLWH. Third, future studies should prioritize emerging agents—such as varenicline, cytisine, and GLP-1 receptor agonists—in PLWH. Key areas include establishing efficacy and safety profiles alongside ART and identifying patient subgroups most likely to benefit from these agents. Fourth, behavioral interventions must address the unique psychosocial stressors faced by PLWH, including stigma, trauma, and economic instability. Fifth, at the health systems level, routine alcohol use and AUD screening in HIV clinical settings represents a critical first step. Provider training remains inadequate, with many HIV clinicians reporting limited confidence in managing alcohol-related problems. The implementation of systems to connect PLWH with AUD treatment could improve outcomes, as could the training of HIV providers in brief alcohol interventions. Sixth, interventions that intentionally strengthen financial stability and economic opportunities for PLWH may have downstream benefits for both HIV and AUD. The heterogeneity in alcohol screening and assessment instruments—many of which are self-reported and non-standardized—limits screening in clinical settings and the generalizability of research findings. More research is needed to optimize existing interventions and develop new ones to improve both UAU and downstream HIV outcomes, as well as to test the best implementation strategies for effective interventions.

## Search Strategy and Selection Criteria

References for this review were identified through systematic searches of PubMed, supported by a medical librarian, Seigel Lori, at the Brown School Library at Washington University in Saint Louis. Additional references were identified through manual searches of article bibliographies and relevant reviews. Specifically, the search included both PubMed subject headings and relevant keywords related to alcohol use and HIV.

1. Alcohol-related terms included:

- *“Alcohol Use Disorder”*,
- *“Alcohol Dependence”*,
- *“Alcohol Abuse”*,
- *“Alcoholism”*,
- *“Binge Drinking”*,
- *“Alcohol-Related Disorders”*,
- *“Problem Drinking”*, and.
- *“Ethanol Abuse”.*

2. HIV-related terms included:

- *“HIV”*,* “HIV Infections”*,
- *“HIV-1”*,* “HIV-2”*,
- *“Acquired immunodeficiency syndrome”*,
- *“AIDS”*,
- *“HIV Long-Term Survivors”*,
- *“HIV Non-Progressors”*,
- *“HIV Seropositivity”*, and.
- the phrase *“living with HIV”*.

A full list of search terms is available upon request. Included in the review are original, peer-reviewed articles that examine the prevalence, correlates, consequences, or treatment of UAU among PLWH. Also included are narrative and systematic reviews, and meta-analysis. Eligible study designs included randomized controlled trials, cohort studies, cross-sectional studies, and qualitative studies. Priority was given to articles published within the last 10 years.

## Data Availability

No datasets were generated or analysed during the current study.
